# Antibacterial properties of antimicrobial peptide HHC36 modified polyetheretherketone

**DOI:** 10.3389/fmicb.2023.1103956

**Published:** 2023-03-14

**Authors:** Weijia Gao, Xiao Han, Duo Sun, Yongli Li, Xiaoli Liu, Shihui Yang, Zhe Zhou, Yuanzheng Qi, Junjie Jiao, Jinghui Zhao

**Affiliations:** ^1^Jilin Provincial Key Laboratory of Tooth Development and Bone Remodeling, Department of Dental Implantology, Hospital of Stomatology, Jilin University, Changchun, Jilin, China; ^2^Jilin Provincial Key Laboratory of Tooth Development and Bone Remodeling, Department of Pediatric Dentistry, Hospital of Stomatology, Jilin University, Changchun, Jilin, China; ^3^Jilin Provincial Key Laboratory of Tooth Development and Bone Remodeling, Department of Prostheses, Hospital of Stomatology, Jilin University, Changchun, Jilin, China

**Keywords:** polyetheretherketone, antimicrobial peptide, surface modification, antibacterial properties, implant

## Abstract

**Introduction:**

Polyetheretherketone (PEEK) is considered to be a new type of orthopedic implant material due to its mechanical properties and biocompatibility. It is becoming a replacement for titanium (Ti) due to its near-human-cortical transmission and modulus of elasticity. However, its clinical application is limited because of its biological inertia and susceptibility to bacterial infection during implantation. To solve this problem, there is an urgent need to improve the antibacterial properties of PEEK implants.

**Methods:**

In this work, we fixed antimicrobial peptide HHC36 on the 3D porous structure of sulfonated PEEK (SPEEK) by a simple solvent evaporation method (HSPEEK), and carried out characterization tests. We evaluated the antibacterial properties and cytocompatibility of the samples *in vitro*. In addition, we evaluated the anti-infection property and biocompatibility of the samples *in vivo* by establishing a rat subcutaneous infection model.

**Results:**

The characterization test results showed that HHC36 was successfully fixed on the surface of SPEEK and released slowly for 10 days. The results of antibacterial experiments *in vitro* showed that HSPEEK could reduce the survival rate of free bacteria, inhibit the growth of bacteria around the sample, and inhibit the formation of biofilm on the sample surface. The cytocompatibility test *in vitro* showed that the sample had no significant effect on the proliferation and viability of L929 cells and had no hemolytic activity on rabbit erythrocytes. *In vivo* experiments, HSPEEK can significantly reduce the bacterial survival rate on the sample surface and the inflammatory reaction in the soft tissue around the sample.

**Discussion:**

We successfully loaded HHC36 onto the surface of SPEEK through a simple solvent evaporation method. The sample has excellent antibacterial properties and good cell compatibility, which can significantly reduce the bacterial survival rate and inflammatory reaction *in vivo*. The above results indicated that we successfully improved the antibacterial property of PEEK by a simple modification strategy, making it a promising material for anti-infection orthopedic implants.

**Figure:**
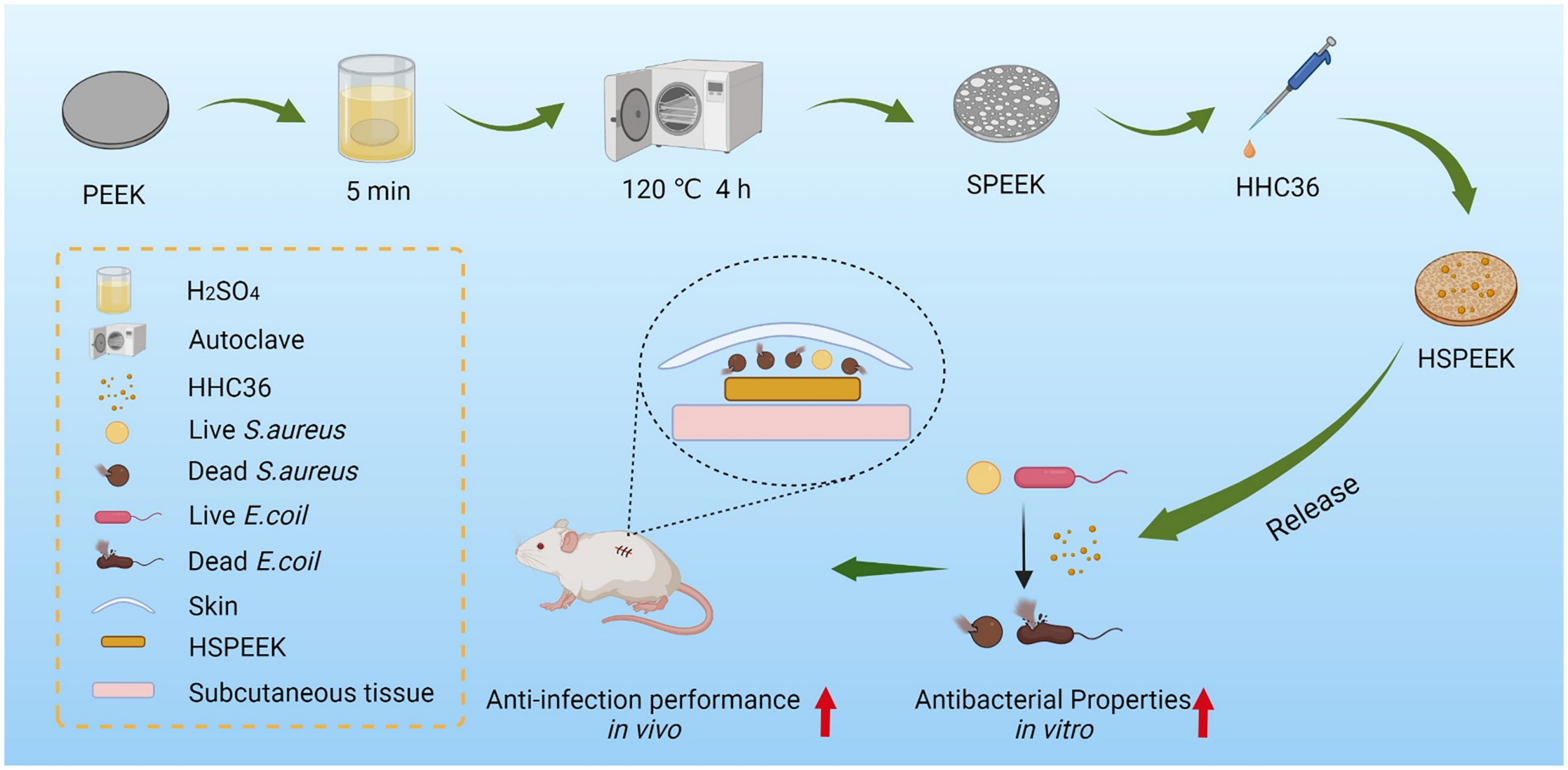
GRAPHICAL ABSTRACT

## 1. Introduction

With the increasing incidence of trauma, joint degeneration and bone tumors, more and more orthopedic implants are being used in orthopedic surgery (Mahyudin et al., 2016). These orthopedic implants play an important role in promoting fracture healing and restoring bone-to-joint anatomy and function. Polyetheretherketone (PEEK) is a high-strength semi-crystalline nonmetallic polymer. The elastic modulus of PEEK is 3–4 Gpa, which is closer to human cortical bone than pure titanium, which can effectively eliminate stress shielding and prevent implants from falling off due to bone absorption (Ma et al., 2021). In addition, PEEK has good chemical stability and does not release harmful by-products such as metal ions. As a non-metallic material, PEEK does not interfere with magnetic resonance imaging (MRI) (Ma et al., 2021). These excellent properties make it a potential substitute for titanium. Orthopedic implants such as intervertebral cages, joint replacement devices and fracture anchors made of PEEK have been gradually applied to clinical practice (Stratton-Powell et al., 2016; He et al., 2021).

Orthopedic implant-related infection (OII) is one of the main causes of orthopedic implant failure, which has destructive effects on patients and health systems. In most cases, it is necessary to remove or revise infected implants for treatment, which will increase the pain and death risk of patients. In addition, it is a huge economic burden to the health system (Nie et al., 2022). Gorth et al. showed that under the same conditions, the biofilm formation rate of PEEK surface was significantly higher than that of Ti, and its affinity for biofilm was 1–6.7 times that of Ti (Gorth et al., 2012). Once free bacteria in the blood or tissue colonize the surface of PEEK, biofilm formation rapidly leads to implant-related infection and eventually implant failure (Croes et al., 2018). In addition, biofilms are difficult to be eliminated by the immune system, and systemic antibiotic therapy is unsatisfactory (Yan and Bassler, 2019). Surgical removal of the implant may lead to disability and impose significant pain and economic burden to patients. Therefore, in order to expand the clinical application of PEEK and make it a better alternative to titanium as an orthopedic implant, it is necessary to improve its antibacterial performance to prevent infection related to PEEK-based implants.

Surface antibacterial modification is a widely used measure of antibacterial modification that can be implemented by preparing antibacterial morphologies or by direct deposition of antibacterial agents on the surface of materials. Many scholars have successfully carried out surface antibacterial modification on different materials, such as stainless steel, glass, metal, cotton fabric, quartz and polyurethane (Xu et al., 2015, 2017, 2018; Guo et al., 2018; Li et al., 2020; Wu et al., 2021), by depositing antibacterial agents directly. In the process of surface antibacterial modification, antibacterial agents play an important role as components to endow the material with antibacterial properties, which usually include antibiotics, naturalextracts, antimicrobial peptides, polymers, metalsandmetaloxides, selenium, fluoride, silicon nitride, graphene oxide and so on (Gao et al., 2022). Among these antibacterial agents, antimicrobial peptides (AMPs) with excellent properties are gradually applied for surface antibacterial modification of biomaterials. AMPs are polypeptides with antibacterial activity, which have positive charge and amphiphilic structure (Yan et al., 2021). Some of them are important components of the innate immunity of most organisms against pathogens, possessing excellent broad-spectrum antibacterial activity and low biological toxicity. AMPs can bind to negatively charged bacterial cell membranes through non-specific electrostatic interactions, leading to cell death by destroying cell membranes. Such antimicrobial mechanisms make it difficult for bacteria to develop resistance. In addition, AMPs have highly selective toxicity to bacterial cell membranes and are not easy to damage mammalian cells (Xi et al., 2016; Zhang et al., 2021). Recently, many studies have confirmed that AMPs could improve the antibacterial properties of biological materials (Pranantyo et al., 2016, 2018, 2019, 2021; Albada and Metzler-Nolte, 2017; Yang et al., 2017; Cao et al., 2018; Zhang et al., 2020; Wang H. et al., 2021; Yan et al., 2021; Jin et al., 2022). HHC36 (KRWWKWWRR) is a highly effective AMP containing nine amino acids predicted and designed by the artificial neural network. It has high antibacterial activity against a variety of multi-resistant “superbugs,” such as MRSA. It exhibits better antibacterial properties than conventional antibiotics (tobramycin, ciprofloxacin, imipenem, ceftazidime) and clinical candidate AMPs (such as MX226 and hLF1-11) (Cherkasov et al., 2009; Kazemzadeh-Narbat et al., 2013). In recent years, some reports have shown that the antibacterial properties of biomaterials have improved after the integration of HHC36 (Lim et al., 2018; Chen et al., 2019, 2020, 2021; Cheng et al., 2021; Guo et al., 2022; Wang et al., 2022).

In this work, we prepared a 3D porous structure on the surface of PEEK by etching with concentrated sulfuric acid, and removed the residual sulfuric acid by hydrothermal treatment. Then, HHC36 was immobilized on the sulfonated and hydrothermal-treated PEEK surface by a simple solvent volatilization method. *Staphylococcus aureus* (*S. aureus*) and *Escherichia coli* (*E. coli*) were used to evaluate the *in vitro* antibacterial properties of the samples. Mouse fibroblast cells (L929) and rabbit erythrocytes were used for *in vitro* cytocompatibility evaluation of samples. In addition, a rat subcutaneous infection model was constructed to evaluate the *in vivo* anti-infection performance and biocompatibility of the samples.

## 2. Materials and methods

### 2.1. Sample preparation

The disk-shaped Ti and PEEK (10 mm × 1 mm) were ultrasonically washed in acetone, ethanol and ultra pure water after being polished with several grades of silicon carbide sandpaper (800 #, 1,200 #, 2000 # and 5,000 #). The dried PEEK disks were placed in conical flasks containing 98% concentrated sulfuric acid and magnetically stirred for 5 min, and then put into deionized water to terminate the reaction. A hydrothermal treatment was then performed at 120°C for 4 h to remove residual sulfuric acid from the surface, and the treated PEEK was designated SPEEK. Subsequently, 50 μL of a 1.5 mg/mL solution of HHC36 in ethanol (KRWWKWWRR, 98.91% pure lyophilized powder, chinapeptides Co, Ltd., China) was applied dropwise to SPEEK and gently air-dried. The loading process was performed in a sterile environment and repeated 20 times. After the HHC36 loading was complete, the samples were gently rinsed three times with Phosphate buffered solution (PBS, Solarbio, China) to remove loosely adhered peptides from the surface. The SPEEK that loaded HHC36 was named HSPEEK. Using the same procedure, HHC36 was loaded onto the untreated PEEK surface. The PEEK that loaded HHC36 was named HPEEK.

### 2.2. Sample characterization

Field emission scanning electron microscope (FESEM, Hitachi S-4800, Japan) and energy dispersive X-ray spectrometer (EDS, Genesis2000, AMETEK, United States) were used to obtain FESEM images and EDS spectra of Ti, PEEK, SPEEK and HSPEEK. A contact angle meter (DSA25, KRUSS,Germany) was utilized to evaluate the surface hydrophilicity or hydrophobicity of the samples. Ultrapure water (5 μL) was dropped on the surface of the sample, and a digital camera was used to take photographs and record the contact angle.

### 2.3. Release profile of AMP HHC36

One HSPEEK and one HPEEK were immersed in 10 ml of PBS at pH 7.4 respectively, and incubated at 37°C with constant shaking (30 rpm/min) for different times (4, 12, 24, 48, 72, 120, and 168 h). 0.3 mL of the supernatant was collected at each time point and subsequently supplemented with PBS to keep the total volume of solution constant. Then, the HHC36 content in the supernatant was detected with a BCA protein assay kit (BCA, Biosharp, China). To determine the total amount of HSPEEK-loaded HHC36, one HSPEEK was sonicated in 2% Sodium dodecyl sulfate (SDS, Solarbio, China) solution for 2 h in reference to Shen et al. (2019). After centrifugation for 5 min (12,000 rpm/min), the supernatant was assayed for HHC36 using the BCA protein assay kit. Each experiment was repeated 3 times.

### 2.4. *In vitro* antibacterial experiments

#### 2.4.1. Bacterial preparation

Single colony of *S. aureus* (ATCC 29213) and *E. coli* (ATCC 25922) were separated by plate scribing respectively, and then the single colony was added to 3 mL of LB liquid medium and shaken overnight at 37°C (150 rpm/min). Subsequently, 100 μL of each bacteria suspension was added to 6 mL of LB liquid medium. To obtain bacteria in the logarithmic growth period, the bacteria suspension was incubated at 37°C for 3 h, so that its OD_600nm_ was between 0.6 and 1.0. The concentration of bacterial suspension was determined by the plate counting method, and the bacterial concentration required for subsequent experiments was obtained by appropriately diluting the bacterial suspension.

#### 2.4.2. Bacterial morphology observation

Ti, PEEK, SPEEK, and HSPEEK were placed in a 24-well plate, and 1 mL of a 1 × 10^7^ CFU/mL suspension of *S. aureus* and *E. coli* was added to each well and incubated at 37°C for 24 h. The samples were taken out and rinsed 3 times with sterile PBS to remove non-adherent bacteria from the samples’ surface. The samples of each group were then placed in 2.5% glutaraldehyde stationary liquid and fixed at 4°C for 8 h. The samples of each group were successively placed in ethanol solution (30, 40, 50, 60, 70, 80, 90, 95, and 100%) for gradient dehydration, with each gradient dehydration lasting for 15 min. Then the samples were dried and sprayed with gold. Finally, the morphology of bacteria adhering to the surface of the samples was observed by FESEM.

#### 2.4.3. Bacterial counting assay

Ti, PEEK, SPEEK and HSPEEK were placed in test tubes and 2 mL of 1 × 10^7^ CFU/mL suspension of *S. aureus* and *E. coli* was added to each tube. After incubation at 37°C for 24 h, the turbidity of the bacterial suspension was recorded by photography, and the OD_600nm_ of the bacterial suspension was measured using an enzyme-labeled instrument (BL340, Biotech, United States). The bacterial suspension was then appropriately diluted with PBS, and 100 μL of the diluted bacterial suspension was uniformly coated on LB plates. After incubation at 37°C for 24 h, the bacterial viability rate was calculated using the following formula: $P\left(\%\right)=A÷B\times 100\%$ , where A represents the average number of colonies on PEEK, SPEEK and HSPEEK, and B represents the average number of colonies on Ti.

#### 2.4.4. Agar diffusion assay

100 μl of *S. aureus* and *E. coli* suspension with the concentration of 1 × 10^8^ CFU/mL were evenly spread on LB solid medium. Ti, PEEK, SPEEK and HSPEEK were uniformly arranged on the surface of the solid medium. After incubation at 37°C for 24 h, the ability to inhibit bacterial growth was evaluated by generally observing the width of the inhibitory band around the samples.

#### 2.4.5. Live/dead bacterial assay

Ti, PEEK, SPEEK and HSPEEK were placed in a 24-well plate, and 1 mL of a 1 × 10^7^ CFU/mL suspension of *S. aureus* and *E. coli* was added to each well. After incubation at 37°C for 48 h, samples were gently washed 3 times with PBS. Fluorescent staining was then performed using a live/dead bacterial staining kit (BBcellProbe, China) with the addition of 300 μL per well mixed solution containing 0.2% N01 and 0.5% propidium iodide (PI) solution and then incubated for 30 min at 37°C in the dark. Finally, the results were observed under a confocal laser scanning microscope (Olympus, Japan).

#### 2.4.6. MTT assay

Ti, PEEK, SPEEK and HSPEEK were placed in a 24-well plate, 1 mL of a 1 × 10^7^ CFU/mL suspension of *S. aureus* and *E. coli* was added to each well, and after incubation at 37°C for 48 h, the samples were transferred to a new 24-well plate and washed with PBS. Then, 1 mL of MTT (Biosharp, China) solution was added to each well and incubated at 37°C for 1.5 h. Subsequently, an equal amount of dimethyl sulfoxide (DMSO, Solarbio, China) was added and incubated on a shaker in the dark for 20 min (100 rpm/min). Finally, the solution was collected, and the OD_540 nm_ was detected by an ELISA reader (Bio-Tek, United States).

### 2.5. *In vitro* cytological experiments

#### 2.5.1. Cell culture

L929 cells (Jilin Key Laboratory of Dental Development and Bone Reconstruction, Changchun, China) were cultured in H-DMEM (Hyclone, United States) at 37°C and 5% CO_2_ atmosphere, supplemented with 10% fetal bovine serum (FBS, GIBCO, United States) and 1% penicillin streptomycin (Penicillin Streptomycin Solution, Hyclone, United States). L929 cells were passaged by pancreatin after reaching 80% confluence.

#### 2.5.2. Live/dead cell assay

The toxicity of the extracts of each sample to L929 cells was evaluated by cell live/dead staining. The extracts of Ti, PEEK, SPEEK and HSPEEK were prepared according to the standard of Biological evaluation of medical devices-Part 12: Sample preparation and reference materials (ISO 10993-12: 2012, IDT). All samples were sterilized by ultraviolet irradiation for 1 h, and then turned over after 30 min of irradiation. The sample was then transferred to a new centrifuge tube and extracted with H-DMEM containing 10% fetal bovine serum at a ratio of 3 cm^2^/mL. The samples were put into a CO_2_ constant temperature cell culture incubator at 37°C for 24 h to prepare the extract. L929 cells at a density of 2 × 10^3^ cells per well were then seeded in a 96-well plate. After 24 h of incubation, the supernatant was discarded, and 100 μL extracts were incubated again for 24 h. The live/dead assay was performed using a calcein AM/PI double staining kit (Beyotime, China). The cells were observed using a fluorescence microscope (Olympus, Japan). Live cells showing green fluorescence were stained only by calcein AM, whereas dead cells showing red fluorescence were stained only by PI.

#### 2.5.3. CCK8 assay

The Cell Counting Kit-8 (CCK8, Biosharp, China) was used to evaluate the effect of Ti, PEEK, SPEEK and HSPEEK extracts on L929 cells proliferation on 1, 3, and 5 d. L929 cells at a density of 2 × 10^3^ cells per well were inoculated in a 96-well plate and the supernatant was discarded after incubation at 37°C for 24 h. Then 100 μL extract of the dip was added to each well and continued to culture until 1, 3, and 5 d. At each time point, H-DMEM/CCK8 premixed solution with a volume ratio of 10:1 was used to replace the culture medium. After incubation at 37°C for 1 h, 100 μL of each group was transferred to a 96-well plate. The OD_450nm_ was measured using an enzyme-labeled instrument (BL340, Biotech, United States).

#### 2.5.4. Haemolysis test

The hemolytic activity of samples in each group was evaluated using rabbit red blood cells. 8 mL of blood was collected from the rabbit ear vein, anticoagulated with heparin, and then diluted in 10 mL of 0.9% normal saline (NS). Ti, PEEK, SPEEK, and HSPEEK were added to diluted rabbit blood containing anticoagulant, in which normal saline was the negative control group (0% hemolysis), and distilled water (DW) was the positive control group (100% hemolysis). The above test tubes were placed in a thermostatic water bath at a temperature of 37°C for 30 min. 0.2 mL diluted anticoagulant rabbit blood was then added to each tube, mixed and placed in a thermostatic water bath for 60 min. After 1,500 rpm/min centrifugation for 5 min, the degree of hemolysis was recorded by a camera. Then, 100 μL supernatant was taken out of each tube and placed in a 96-well plate, and hemoglobin release from the supernatant was detected using a microplate reader at OD_545nm_.

$\mathrm{Hemolyticrate}\left(\%\right)=\frac{OD_{\mathrm{samples}}-OD_{\mathrm{negative}}}{OD_{\mathrm{positive}}-OD_{\mathrm{negative}}}\times 100\%$ ,Where samples represent Ti, PEEK, SPEEK, and HSPEEK. Negative represents NS, and positive represents DW.

### 2.6. Animal experiments

#### 2.6.1. Establishment of rat subcutaneous infection model

The experiment was approved by the Institutional Animal Care and Use Committee of Jilin University (No. 20220456). For the rat subcutaneous infection model, 16 Wistar rats (4 rats per group for Ti, PEEK, SPEEK, and HSPEEK) were used. All surgical instruments are high-temperature and high-pressure sterilized. The back hair of these rats was shaved off preoperatively and disinfected with povidone iodine. Intraoperatively, they were anesthetized by inhalation of 1.5% isopentane, and a 1 cm incision was made on the back, after which the samples (10 mm × 1 mm) were inserted subcutaneously. The skin incision was then carefully sutured. Finally, 100 μl dilution of *S. aureus* (1 × 10^7^ CFU/mL) was injected into the subcutaneous sample surface by a microinjector.

#### 2.6.2. *In vivo* anti-infection performance evaluation

After 7 d of implantation, the samples under the skin were removed and soaked in PBS. The test tube containing the samples was vortexed for 30 s and sonicated for 10 min to allow bacteria adhering to the sample surface to fall off. PBS containing bacteria was appropriately diluted and plated on agar plates for incubation at 37°C for 24 h. The bacterial viability rate was calculated using the formula: $P\left(\%\right)=A÷B\times 100\%$ , where A represents the average number of colonies on PEEK, SPEEK and HSPEEK samples, and B represents the average number of colonies on Ti samples.

#### 2.6.3. *In vivo* biocompatibility evaluation

After the removal of implants, the skin around the samples was collected and fixed in paraformaldehyde. Skin tissue was subsequently embedded in paraffin for histological sectioning. H&E staining was used to stain inflammatory cells in skin tissue. Representative images were taken using a light microscope (Olympus, Japan). The inflammatory cells were counted by ImageJ software, and the biocompatibility of each group was evaluated according to the density of inflammatory cells.

### 2.7. Statistical analysis

All data are expressed as means ± standard deviation (SD) from triplicate independent experiments. One-way analysis of variance (ANOVA) and Tukey’s multiple comparison tests were used to evaluate the statistically significant differences among groups. All statistical analyses were performed using SPSS 19.0 software (SPSS, Chicago, IL, United States). ^*^p < 0.05, ^**^p < 0.01, and ^***^p < 0.001. All experiments were performed in triplicate and repeated at least three times.

## 3. Results

### 3.1. Sample characterization

**Figure 1:**
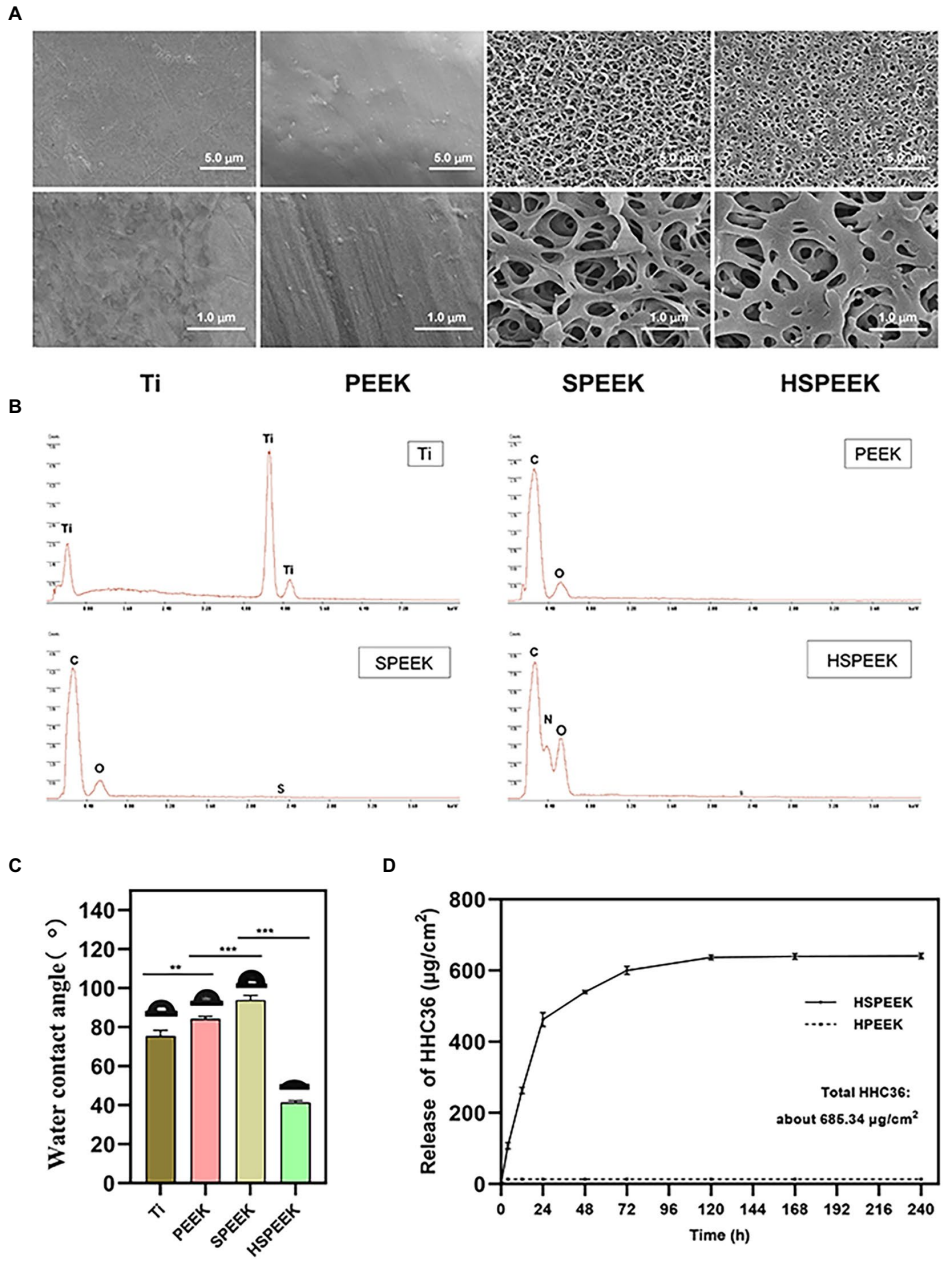
**(A)** FESEM morphology at low and high power of Ti, PEEK, SPEEK and HSPEEK, **(B)** EDS spectra of Ti, PEEK, SPEEK and HSPEEK, **(C)** Water contact angles of Ti, PEEK, SPEEK and HSPEEK, and **(D)** Release of HHC36 from HSPEEK and HPEEK in PBS. (*n* = 3, ^**^p < 0.01, ^***^p < 0.001).

The surface microstructure and chemical composition of the samples were examined by FESEM and EDS. FESEM images showed that Ti has a relatively flat surface. PEEK is slightly rougher than Ti, and slight protrusions could be observed. Compared with relatively flat Ti and PEEK, a 3D network structure could be observed on the surface of SPEEK (Figure 1A). The pore size of HSPEEK is significantly smaller than that of SPEEK, which may result from the loading of HHC36 (Yuan et al., 2019). Nitrogen peaks were identified by EDS on the HSPEEK surface (Figure 1B), indicating that HHC36 was successfully immobilized on the HSPEEK surface. The water contact angle was used to evaluate the hydrophilicity or hydrophobicity of the sample surface (Figure 1C). The water contact angle of Ti was 75.60° ± 2.81°. The water contact angle of PEEK was 84.33° ± 1.29°, which illustrates that PEEK is more hydrophobic than titanium (*p* < 0.01). After sulfonation and hydrothermal reaction, the water contact angle of SPEEK increased to 93.90° ± 2.43°and became more hydrophobic (*p* < 0.001). After loading HHC36, the water contact angle of HSPEEK decreased significantly to 41.47° ± 0.85° and became hydrophilic (*p* < 0.001), which may result from the hydrophilic residues in HHC36 (Wang et al., 2022).

### 3.2. Release profile of AMP HHC36

HHC36 was eluted from the HSPEEK using SDS solution under sonication, and the actual total loading of HHC36 was measured to be 685.34 μg/cm^2^. According to the actual loading capacity, it could be calculated that the cumulative release rate of HHC36 was 71.15% at 24 h and 92.39% at 72 h. As shown in Figure 1D, HHC36 on the HSPEEK was slowly released until 10 d after an initial fast release. For HPEEK, only trace amounts of HHC36 remained on its surface and no sustained release was observed. Since the loading of HHC36 required 20 cycles, the theoretical total loading after 20 loading cycles was further calculated to be 954.93 μg/cm^2^, and the loading efficiency was 71.82%, which might be due to the loss of HHC36 during the loading process.

### 3.3. *In vitro* antibacterial experiments

**Figure 2:**
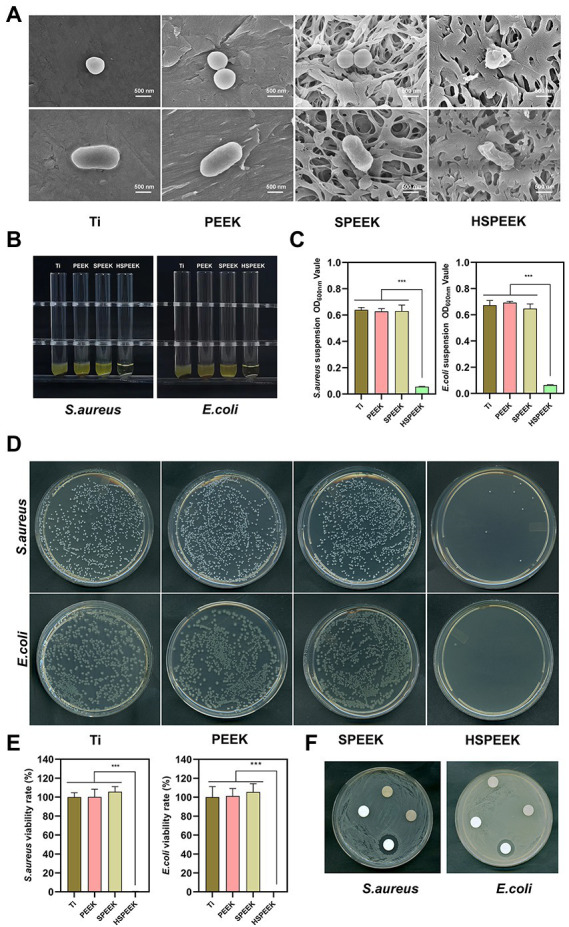
**(A)** Morphology of *S. aureus* and *E. coli* after 24 h coculture with Ti, PEEK, SPEEK and HSPEEK, **(B)** Photos of the turbidity of the bacterial suspension after 24 h of coculture with each group of samples, **(C)** The OD_600nm_ value of the bacterial suspension, **(D)** Typical images of colonies incubated for 24 h with each group of samples, **(E)** Viability rate of *S. aureus* and *E. coli*, and **(F)** The bacterial inhibition zone of each group of samples. (*n* = 3, ^***^p < 0.001).

The effects on the cell morphology of *S. aureus* and *E. coli* on different samples were determined by FESEM. As shown in Figure 2A, the two bacteria on the surfaces of Ti, PEEK and SPEEK were complete. Two bacteria on the HSPEEK surface became severely distorted. These results indicated that HHC36 could destroy the cell membrane integrity of *S. aureus* and *E. coli*, and Ti, PEEK and SPEEK did not possess this ability.

The effect of each group of samples on the turbidity of *S. aureus* and *E. coli* suspensions could be seen in Figure 2B. The suspensions around Ti, PEEK and SPEEK were turbid. In contrast, the suspensions around HSPEEK were relatively clear. Figure 2C showed that the OD_600nm_ of bacterial suspension in HSPEEK group was significantly lower than that of Ti, PEEK and SPEEK groups (*p* < 0.001), while there was no significant difference among Ti, PEEK and SPEEK groups (*p* > 0.05).

As shown in Figure 2D, the bacterial colonies on Ti, PEEK, and SPEEK covered almost the entire solid medium, with several colonies observed in HSPEEK. Figure 2E showed that the bacterial viability rate of HSEEK was significantly lower than that of Ti, PEEK and SPEEK (*p* < 0.001), and the bacterial viability rate of the *S. aureus* and *E. coli* suspension cocultured with HSEEK was less than 1%. The bacterial viability rates of Ti, PEEK and SPEEK were close to 100%, and there was no significant difference among the three groups (*p* > 0.05), suggesting that Ti, PEEK, and SPEEK had no antibacterial activity against *S. aureus* and *E. coli* suspension, which was consistent with the turbid condition of the bacterial suspension.

The agar diffusion test showed a prominent inhibition zone around the HHC36 immobilized sample (Figure 2F). There was no bacterial growth in this area, indicating that HHC36 could be released from the porous structure and inhibited the growth of surrounding bacteria. In addition, there was no inhibition zone around Ti, PEEK, and SPEEK, indicating that they did not possess the ability to inhibit the growth of the surrounding bacteria.

**Figure 3:**
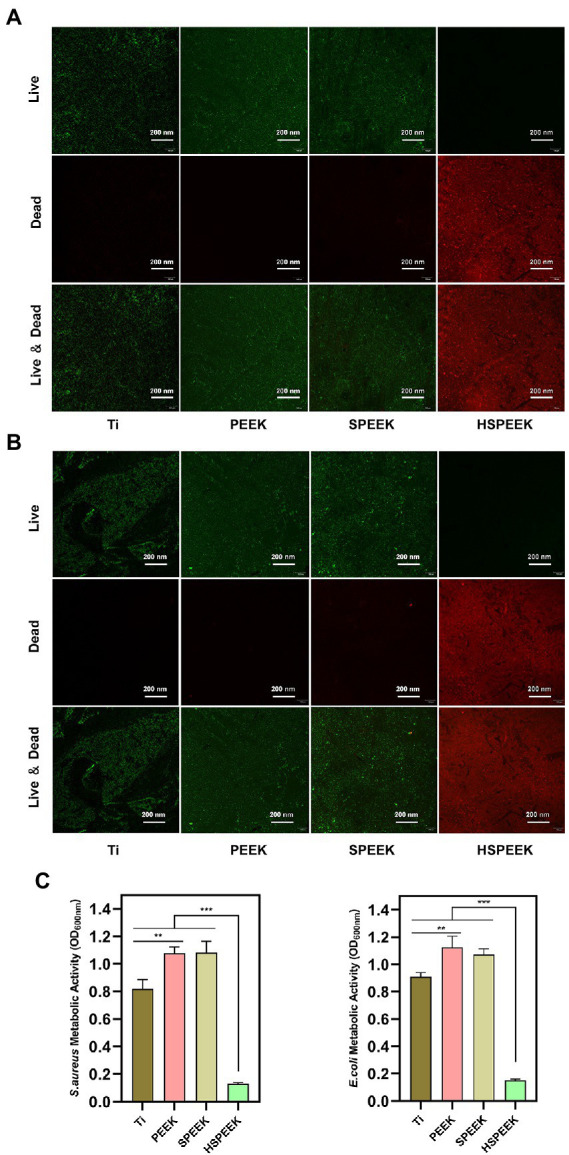
**(A)** Live/dead staining image of *S. aureus* biofilm after 48 h coculture with Ti, PEEK, SPEEK and HSPEEK, **(B)** Live/dead staining image of *E. coli* biofilm after 48 h coculture with Ti, PEEK, SPEEK and HSPEEK, and **(C)** Metabolic activity of *S. aureus* and *E. coli* biofilms after 48 h coculture on the surface of samples from each group. (*n* = 3, ^**^p < 0.01, ^***^p < 0.001).

Effects of samples in each group on *E. coli* and *S. aureus* biofilms as shown in Figures 3A,B, live bacteria were stained green fluorescence by N01, and dead bacteria were stained red fluorescence by PI. The surface of HSPEEK showed red fluorescence and no green fluorescence, which illustrated that its surface was almost covered by dead bacteria without living bacteria, implying that HSPEEK could prevent biofilm formation by killing bacteria. The surfaces of Ti, PEEK and SPEEK were almost all covered by viable bacteria with green fluorescence. At the same time, there was no red area, indicating that they did not have any antibacterial activity.

The results of MTT analysis were shown in Figure 3C. The biofilm metabolic activities of both *S. aureus* and *E. coli* in the HSPEEK group were significantly lower than those in the other groups (*p* < 0.001), indicating that HSPEEK could significantly reduce the metabolic activity of bacterial biofilms. In addition, it was found that the biofilm metabolic activity of the Ti surface was significantly lower than that of PEEK (*p* < 0.05).

### 3.4. *In vitro* cytological experiments

**Figure 4:**
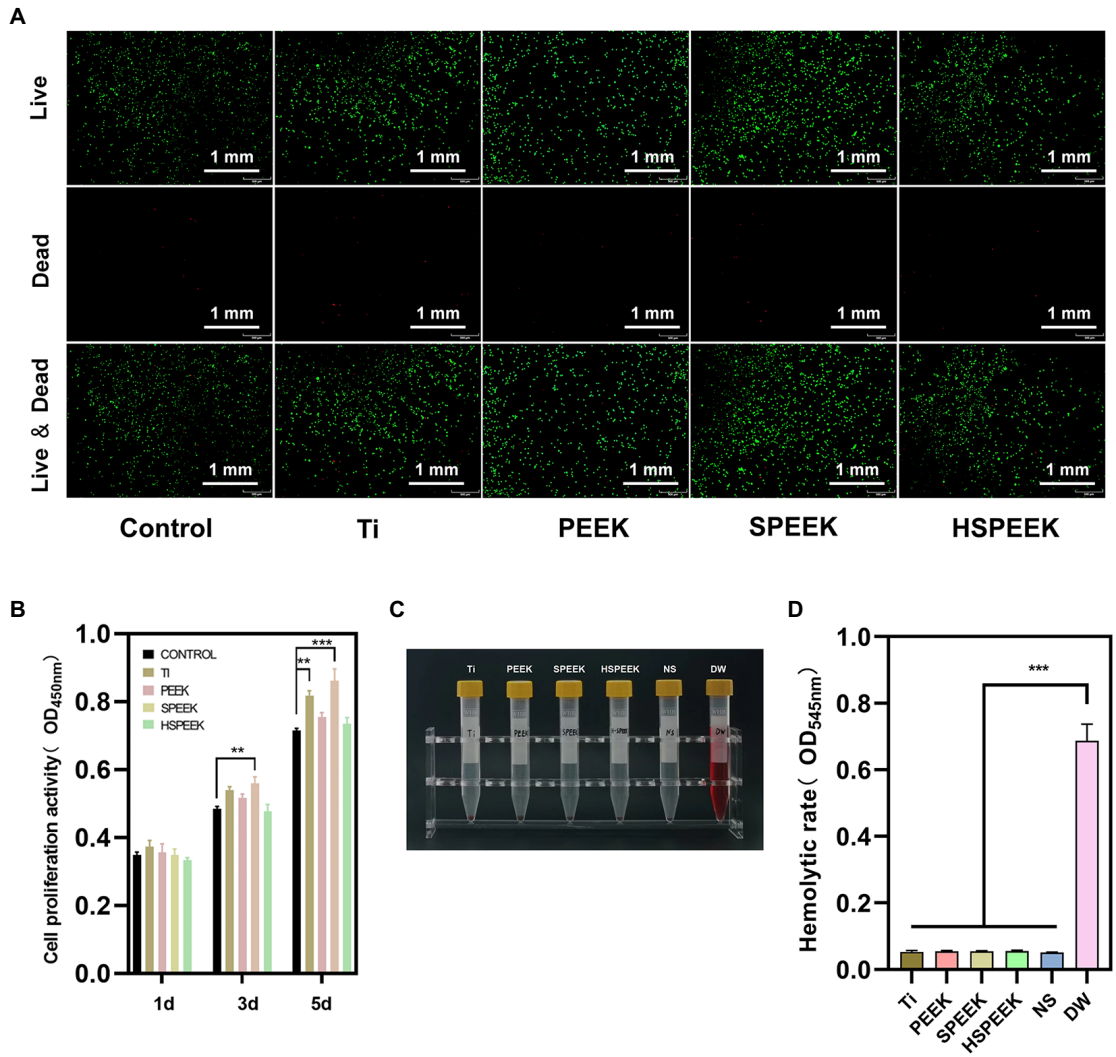
**(A)** Live/dead staining images of L929 cells after coculture with Ti, PEEK, SPEEK and HSPEEK extracts for 24 h, **(B)** The proliferation of L929 cells after 1, 3 and 5 d of coculture with sample extracts from each group, **(C)** Photographs of rabbit blood supernatant after 1 h of incubation with samples from each group, and **(D)** Hemolytic rate of samples from each group. (*n* = 3, ^**^p < 0.01, ^***^p < 0.001).

The toxicity of the extracts of the samples to L929 cells was determined by live/dead staining. The results showed that a large number of living cells emitting green fluorescence could be seen in all groups, while it was difficult to find dead cells emitting red fluorescence (Figure 4A). The results showed that the extracts of Ti, PEEK, SPEEK, and HSPEEK had almost no cytotoxicity.

The effect of each group on cell proliferation was confirmed by measurement of CCK8 assay, and the results was shown in Figure 4B. The cell proliferative viability of the Ti group was close to or slightly higher than that of the control group on 1 d and 3 d, and significantly higher than the control group on 5 d (*p* < 0.01), indicating that Ti could promote cell proliferation as time progresses. The cell proliferative activity of PEEK was always slightly higher than that of the control group at each time point, still no significant differences were observed (*p* > 0.05), suggesting that PEEK did not significantly promote cell proliferation. The cell proliferative activity of SPEEK was significantly higher on 3 d than the control group (*p* < 0.01), and this trend was more pronounced on 5 d (*p* < 0.001). This phenomenon may be related to the fact that 3D network structures can promote cell proliferation (Ouyang et al., 2016). Compared with the control group, the cell proliferative activity of HSPEEK was not significantly different at each time point (*p* > 0.05), proving that HSPEEK had no significant effect on cell proliferation.

In the hemolysis test, obvious hemolysis in the positive control group could be observed (Figure 4C). Ti, PEEK, SPEEK, HSPEEK and the negative control group had no macroscopic hemolysis, and the hemolytic rate was less than 5% (Figure 4D), indicating that all samples had low hemolytic activity.

### 3.5. Animal experiments

#### 3.5.1. *In vivo* anti-infection performance evaluation

**Figure 5:**
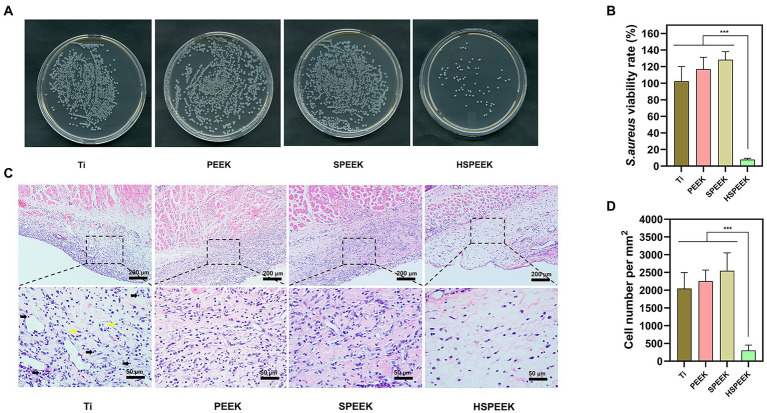
**(A)** Images of *S. aureus* colonies on the surface of Ti, PEEK, SPEEK and HSPEEK, **(B)** *S. aureus* viability rate on the surface of each group of samples, **(C)** H&E staining results of soft tissue surrounding each group of samples. Black arrows represent inflammatory cells and yellow arrows represent healthy tissue cells, and **(D)** The density of inflammatory cells in the surrounding soft tissue of each group of samples. (*n* = 4, ^***^p < 0.001).

Figure 5A showed that the bacterial colonies on Ti, PEEK and SPEEK covered most of the solid medium, and only a few colonies were found in the HSPEEK. Figure 5B showed the bacterial viability rate on the surface of samples in each group. No significant differences in bacterial viability were observed for the Ti, PEEK, and SPEEK surfaces (*p* > 0.05). The bacterial viability rate of 7.62% in the HSPEEK group was significantly lower than that of the other groups (*p* < 0.001), indicating that HSPEEK could significantly reduce the number of *S. aureus* on its surface, which was consistent with the results of the *in vitro* experiments.

#### 3.5.2. *In vivo* biocompatibility evaluation

Figure 5C showed the severe inflammatory response of the skin in contact with the surfaces of Ti, PEEK, and SPEEK, where a large infiltration of inflammatory cells was observed. Skin in contact with the HSPEEK surface had only a few inflammatory cells. Figure 5D showed that the density of inflammatory cells in the skin in contact with the HSPEEK surface was significantly lower than Ti, PEEK, and SPEEK groups (*p* < 0.001), suggesting that HSPEEK reduced not only the number of bacteria but also the inflammatory response caused by bacteria.

## 4. Discussion

In this work, HHC36 was added to SPEEK by simple sulfonation technique, hydrothermal treatment and solvent volatilization method. The sulfonation process is a simple and controllable modification technology. In industry, sulfonation treatment of PEEK has been used to produce SPEEK membranes with excellent proton conductivity, and it is mainly used in fuel cells (Montero et al., 2017). Sulfonation treatment of PEEK can also produce desiccants with porous structures whose surface micropores contribute to the adsorption of moisture in humid air (Akhtar et al., 2021). Traditionally, sulfonation reactions require a long time at high temperature (Yee et al., 2013). However, Zhao et al. found that SPEEK surfaces modified by short-time sulfonation (5 min under ultrasonic stirring) have shown biocompatible (Zhao et al., 2013). The sulfonation reaction could form a 3D network structure on the surface of the polymer and serve as a drug loading platform to play a role in bone formation, anti-inflammation and anti-bacteria (Sun et al., 2018; Wang et al., 2019; Zhu et al., 2019; Wang et al., 2020; Wang X. J. et al., 2021; Yu et al., 2021a,b; Zheng et al., 2022). There have been many researchers sulfonating the surface of PEEK and utilizing its porous structure to load antimicrobial agents, endowing PEEK with antimicrobial properties (He et al., 2019; Yang et al., 2019, 2021; Yuan et al., 2019). In addition to the porous structure, the electrostatic attraction also contributed to the loading of HHC36 on the surface of SPEEK. HHC36 is a polypeptide with 5 positive charges, an isoelectric point of 12.31, and is highly positive at pH 7.4. Kazemzadeh-Narba et al. found that HHC36 could be loaded on calcium phosphate (CaP) coating with porous structures, and the positively charged residues of HHC36 and negatively charged phosphate groups of CaP had an electrostatic affinity, which to some extent conferred sustained release HHC36 from CaP coating (Kazemzadeh-Narbat et al., 2012). Ouyang et al. measured the Zeta potential of PEEK surface after sulfonation and hydrothermal reaction treatment and found that its surface was negatively charged (Ouyang et al., 2016). We speculate that similar electrostatic affinities exist between positively charged residues in HHC36 and negatively charged sulfonic acid groups on the surface of SPEEK, which may help explain why HHC36 was still released gently up to 10 d after an initial burst release. Based on the unique release profile of HSPEEK, HHC36 could be released rapidly early in the procedure to control infection and then slowly to prevent potential infection.

Bacterial infection is considered to be an important factor leading to implant failure. During orthopedic surgery, implants are susceptible to contamination by bacteria in the surrounding environment. Planktonic bacteria adhere to the surface of the implant and rapidly evolve into the biofilm. Once the biofilm on the surface of the implant matures, it will be difficult for the human immune system and external antibiotics to eliminate them, which will lead to implant failure (Alonso et al., 2020; Escobar et al., 2020; Jalil et al., 2020). Therefore, inhibiting biofilm formation by killing plankton bacteria is an effective measure to prevent bacterial infection. *In vitro* antibacterial tests showed that HSPEEK killed more than 99% of the bacteria in suspensions of *S. aureus* and *E. coli*. The result was consistent with the clarity and OD_600nm_ of the bacterial suspension surrounding the HSPEEK group. The death of bacterioplankton could be attributed to the effect of HHC36 on bacterial membrane destruction. In this work, FESEM results showed that HHC36 could effectively destroy the integrity of *S. aureus* and *E. coli*, which might cause the outflow of bacterial contents and death. Chen et al. monitored the binding of HHC36 to bacteria in real time using aggregation-induced emission probes. They observed the aggregation of HHC36 on bacterial membranes and the destruction of membrane structure, which resulted in the subsequent efflux of nucleic acids or protein from within the bacteria (Chen et al., 2018). Some scholars found that HHC36 has the ability to disrupt the integrity of bacterial cell membranes through FESEM (Chen et al., 2021). HHC36 has this non-specific antibacterial mechanism that destroys cell membranes so it may have advantages over traditional antibiotics against resistant bacteria such as MARS (Chen et al., 2020, 2021; Guo et al., 2022). Reducing the bacterial viability in the bacterial suspension may inhibit the formation of bacterial biofilm on the material surface. The results of bacterial live/dead staining and MTT assay supported this view. The HSPEEK surface is covered with dead bacteria and almost no live bacteria. MTT assay showed that HHC36 significantly reduced the metabolic activity of biofilm and the formation of biofilm. It could be noted that the biofilm metabolic activity of the Ti surface was significantly lower than that of PEEK and SPEEK, which might be related to the lower affinity of titanium for biofilm than that of PEEK (Gorth et al., 2012; Krätzig et al., 2021).

An ideal orthopedic implant should not only have excellent antibacterial capacity but also good cytocompatibility. Cell live/dead staining and CCK8 assay showed that HSPEEK extract was non-toxic to L929 cells and did not negatively affect cell proliferation. It also showed low hemolytic activity, which was not different from other groups, demonstrating the good cytocompatibility of HHC36. The good cytocompatibility of HSPEEK is related to the unique antibacterial mechanism of AMPs. Unlike bacterial cell membranes with large amounts of negatively charged phospholipids, mammalian cell membranes are only rich in electrically neutral zwitterionic phospholipids and cholesterol, which make AMPs less likely to bind to and cause damage to mammalian cells. HHC36 has been shown to have very low toxicity to mammalian cells (Kazemzadeh-Narbat et al., 2013; He et al., 2018; Miao et al., 2021).

In animal experiments, the bacterial viability rate of the HSPEEK surface was significantly lower than other groups, which was consistent with *in vitro* colony count results. However, there was little difference in bacterial viability between Ti and PEEK. The extent of the inflammatory response in rat skin was almost consistent with the bacterial survival results for each group of materials. Rochford et al. implanted PEEK and Ti into two strains of mice (C57BL/6 and BALB/C). They found that the number of bacteria on the surface of the two materials and the inflammatory response in the surrounding tissue did not show significant differences over time. The findings suggest that the choice of Ti and PEEK has little effect on the progression of infection once implant-associated infection occurs *in vivo* (Rochford et al., 2019). Interestingly, although not significant, the bacterial viability rate on the SPEEK surface was found to be greater than that of Ti and PEEK in this study. This phenomenon may be because SPEEK has a larger surface area, which is conducive to bacterial adhesion. *S. aureus* can escape from the immune system and mislead the immune system, causing serious inflammation (Zhang et al., 2022). H&E staining images showed dense infiltration of inflammatory cells in the soft tissues around Ti, PEEK, SPEEK and HSPEEK, consistent with the high number of *S. aureus* adhering to the surfaces of these samples. HSPEEK had significantly lower inflammatory cell density in the surrounding soft tissue than Ti, PEEK and SPEEK, indicating the good biocompatibility of HSPEEK.

## 5. Conclusion

The AMP HHC36 was immobilized on the 3D porous structure of SPEEK by simple sulfonation technology, hydrothermal treatment and solvent volatilization method. The *in vitro* antibacterial results showed that HSPEEK exhibited excellent antibacterial properties against *S*. *aureus* and *E. coli*, killing plankton bacteria and inhibiting the formation of bacterial biofilm. *In vitro* cytological experiments results indicated that HSPEEK had good cytocompatibility. Moreover, HSPEEK exhibited excellent anti-infection performance and biocompatibility in the rat subcutaneous infection model, reducing not only the bacterial viability rate but also the inflammation. This study provided a new strategy for improving the antibacterial properties of PEEK, which has a broad application prospect in orthopedic implants. The limitation of this study is that the effect of HHC36 on osseointegration of implants has not been evaluated. This evaluation will be carried out in our further study. In addition, how to further improve the biocompatibility of HSPEEK by modification will also be the focus of our future research.

## Data availability statement

The raw data supporting the conclusions of this article will be made available by the authors, without undue reservation.

## Ethics statement

The animal study was reviewed and approved by Institutional Animal Care and Use Committee of Jilin University.

## Author contributions

WG and JZ: conceptualization. XH: methodology. ZZ: software. XH and DS: validation. YL: formal analysis. XL: investigation. SY: resources. WG: data curation and writing—review and editing. DS: writing—original draft preparation. YQ and JJ: visualization. XH and JZ: supervision. JZ: project administration and funding acquisition. All authors contributed to the article and approved the submitted version.

## Funding

This research was funded by Project of Development Plan of Science and Technology of Jilin Province (no. 20200403094SF) and Natural Science Foundation of Jilin Province (no. YDZJ202201ZYTS078).

## Conflict of interest

The authors declare that the research was conducted in the absence of any commercial or financial relationships that could be construed as a potential conflict of interest.

## Publisher’s note

All claims expressed in this article are solely those of the authors and do not necessarily represent those of their affiliated organizations, or those of the publisher, the editors and the reviewers. Any product that may be evaluated in this article, or claim that may be made by its manufacturer, is not guaranteed or endorsed by the publisher.
